# The regulatory role of metabolic organ-secreted factors in the nonalcoholic fatty liver disease and cardiovascular disease

**DOI:** 10.3389/fcvm.2023.1119005

**Published:** 2023-04-26

**Authors:** Li Qin, Junru Wu, Xuejing Sun, Xuewei Huang, Wei Huang, Chunyan Weng, Jingjing Cai

**Affiliations:** Department of Cardiology, The Third Xiangya Hospital of Central South University, Changsha, China

**Keywords:** nonalcoholic fatty liver disease, cardiovascular disease, hepatokines, adipokines, cytokines

## Abstract

Nonalcoholic fatty liver disease (NAFLD) is a chronic metabolic disease characterized by an excessive accumulation of fat in the liver, which is becoming a major global health problem, affecting about a quarter of the population. In the past decade, mounting studies have found that 25%–40% of NAFLD patients have cardiovascular disease (CVD), and CVD is one of the leading causes of death in these subjects. However, it has not attracted enough awareness and emphasis from clinicians, and the underlying mechanisms of CVD in NAFLD patients remain unclear. Available research reveals that inflammation, insulin resistance, oxidative stress, and glucose and lipid metabolism disorders play indispensable roles in the pathogenesis of CVD in NAFLD. Notably, emerging evidence indicates that metabolic organ-secreted factors, including hepatokines, adipokines, cytokines, extracellular vesicles, and gut-derived factors, are also involved in the occurrence and development of metabolic disease and CVD. Nevertheless, few studies have focused on the role of metabolic organ-secreted factors in NAFLD and CVD. Therefore, in this review, we summarize the relationship between metabolic organ-secreted factors and NAFLD as well as CVD, which is beneficial for clinicians to comprehensive and detailed understanding of the association between both diseases and strengthen management to improve adverse cardiovascular prognosis and survival.

## 1. Introduction

Nonalcoholic fatty liver disease (NAFLD) is a chronic progressive liver disease characterized by excessive intrahepatic lipid accumulation associated with metabolic dysfunction in the absence of excessive alcohol drinking and other definite liver-damaging factors (1). The disease spectrum of NAFLD mainly includes simple steatosis (SS), nonalcoholic steatohepatitis (NASH), cirrhosis and hepatocellular carcinoma (HCC). With the dramatic improvement in living standards and the increase in metabolic diseases, the prevalence and incidence of NAFLD are also significantly elevated, which may burden human health. More recently, a meta-analysis of the global epidemic of NAFLD indicated that the global prevalence of this disease was 25.24%, and the incidence rate ranged from 28.01 per 1,000 to 52.34 per 1,000 person-years (2). Moreover, the prevalence of NAFLD varied by region, age, gender, ethnicity, and economic condition (2). A growing body of evidence demonstrated that the complications of NAFLD can not only be involved in advanced liver disease (cirrhosis and HCC) but also contain severe extrahepatic diseases, especially cardiovascular diseases (CVD) and extrahepatic malignancies (1). CVD was not uncommon in patients with NAFLD, and its prevalence was reported to be about 25%–40% (3). Besides, CVD was the leading cause of mortality in NAFLD patients, accounting for 40%–50% of all deaths (4). Mantovani et al. found that NAFLD patients had an increased risk of fatal and non-fatal CVD events [hazard ratio (HR) = 1.45], and this risk further increased with the severity of NAFLD (5). However, the underlying pathogenesis of CVD in patients with NAFLD is not completely elucidated nowadays. Mounting evidence revealed that NAFLD might directly or indirectly promote the occurrence and development of CVD through a variety of mechanisms, including insulin resistance (IR), lipid metabolism disorders, systemic inflammation, oxidative stress, dysregulation of neuroendocrine homeostasis, hypercoagulation state and intestinal dysbiosis (3). Notably, in the past few decades, increasing studies proposed that liver, adipose tissue and gut, as the main secretory organs, can produce some metabolic organ-secreted factors, such as hepatokines, adipokines, cytokines, chemokines, complements, extracellular vesicles and gut-derived factors, which also played a crucial role in the pathogenesis of metabolic diseases and CVD (3). However, few studies systemic and detailed elucidate the effects of metabolic organ-secreted factors in the development of CVD and NAFLD. A comprehensive understanding of the relationship between NAFLD and CVD could be beneficial for improving the prognosis and survival of patients. Therefore, this article reviews the association between metabolic organ-secreted factors secretion and NAFLD as well as their regulatory effects on CVD, which may be helpful for clinicians to early identify the risk of CVD in NAFLD patients and strengthen the management to improve adverse cardiovascular prognosis.

## 2. Metabolic organ-secreted factors in NAFLD

**Figure 1:**
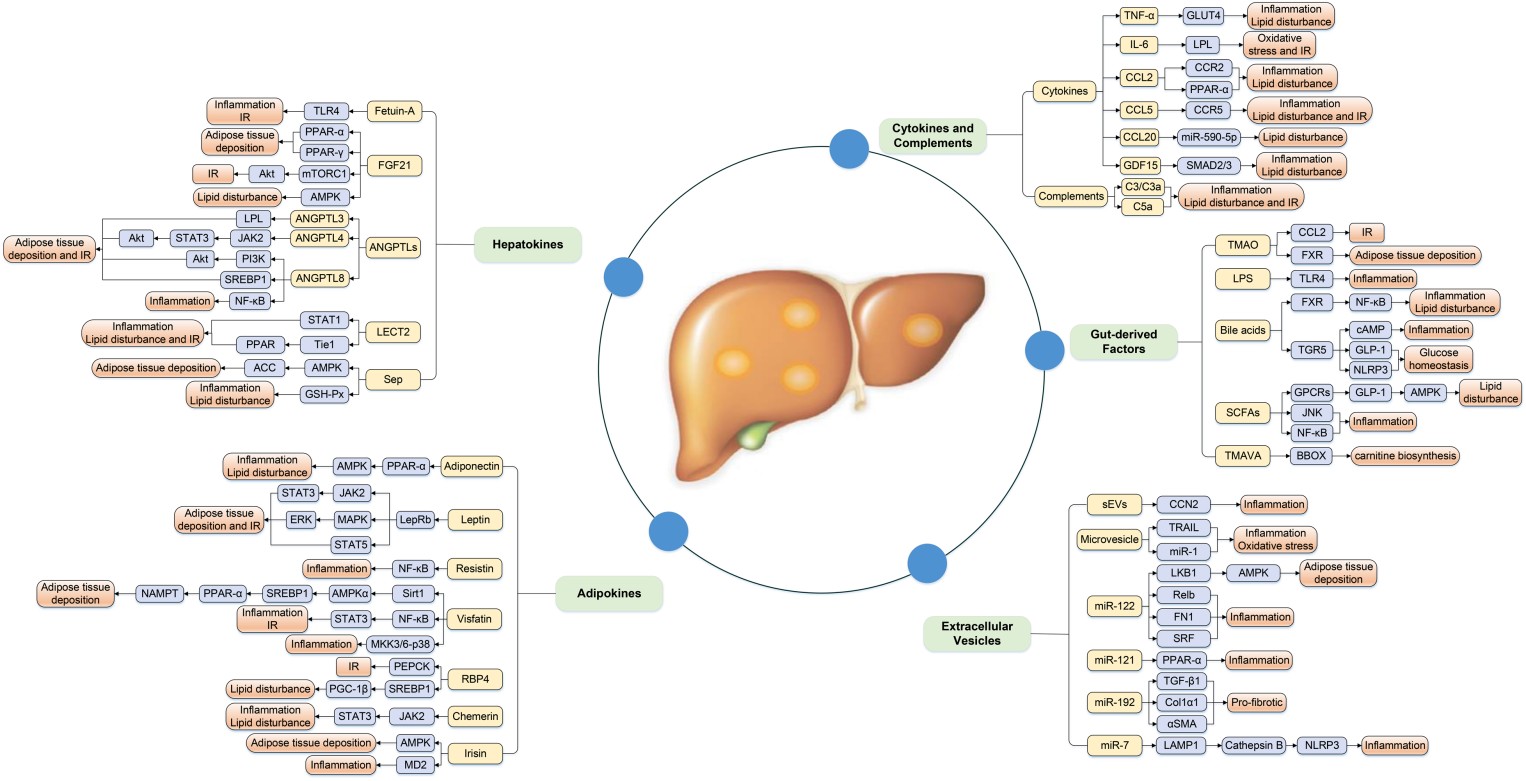
Complex mechanism between metabolic organ-secreted factors and increased risk of NAFLD. Metabolic organ-secreted factors, such as hepatokines, adipokines, cytokines, chemokines, complements, extracellular vesicles and gut-derived factors, promote the pathogenesis of NAFLD via a variety of signaling pathways and mechanisms, including disturbance lipid and glucose metabolism, oxidative stress, chronic inflammation and so on. ACC, acetyl-CoA carboxylase; Akt, protein kinase B; AMPK, adenosine monophosphate-activated protein kinase; ANGPTLs, angiopoietin-like proteins; BBOX, γ-butyrobetaine hydroxylase; CCL2, chemokine ligand 2; CCL5, chemokine ligand 5; CCL20, chemokine ligand 20; CCN2, connective tissue growth factor; Col1α1, collagen type I α 1; ERK, extracellular signal-regulated kinase; EVs, extracellular vesicles; FGF21, fibroblast growth factor 21; FXR, farnesoid × receptor; GDF15, growth differentiation factor 15; GLP-1, glucagon-like peptide-1; GLUT4, glucose transport 4; GPCRs, G-protein-coupled receptors; GSH-Px, glutathione peroxidase; IL-6, interleukin-6; IR, insulin resistance; JAK2, Janus kinase 2; JNK, c-Jun N-terminal kinase; LAMP1, lysosomal associated membrane protein 1; LECT2, leukocyte cell-derived chemotaxin 2; LepRb, isoform b of leptin receptor; LKB1, liver kinase B1; LPL, lipoprotein lipase; LPS, lipopolysaccharide; IR, insulin resistance; MAPK, mitogen-activated protein kinase; MD2, myeloid differentiation factor 2; MKK, MAPK kinase; mTORC1, mammalian target of rapamycin complex 1; NAMPT, nicotinamide phosphoribosyltransferase; NLRP3, NOD-like receptor protein 3; PEPCK, gluconeogenic enzyme phosphoenolpyruvate carboxykinase; PGC-1β, peroxisome proliferator-activated receptor-γ coactivator 1β; PI3K, phosphatidylinositol 3 kinase; PPAR-α, peroxisome proliferator-activated receptor-α; PPAR-γ, peroxisome proliferator-activated receptor-γ; RBP4, retinol binding protein-4; SCFAs, short-chain fatty acids; Sep, selenoprotein P; sEVs, small EVs; αSMA, α-smooth muscle actin; SREBP1, sterol regulatory element-binding protein-1; STAT1, signal transducer and activator of transcription-1; STAT3, signal transducer and activator of transcription-3; STAT5, signal transducer and activator of transcription-5; TGF-β1, transforming growth factor β1; TGR5, Takeda G-protein receptor 5; TLR4, toll-like receptor 4; TMAO, trimethylamine N-oxide; TMAVA, N,N,N-trimethyl-5-aminovaleric acid; TNF-α, tumor necrosis factor-α; TRAIL, TNF-related apoptosis-inducing ligand.

Increasing evidence has illustrated that the association between metabolic organ-secreted factors and increased risk of NAFLD, and the underlying mechanisms may involve a series of processes associated with glucose and lipid metabolism, IR, oxidative stress, and chronic inflammation and so on (Figure 1).

### 2.1. Hepatokines

Hepatokines are a class of proteins produced by hepatocytes, mainly including fetuin-A, fibroblast growth factor 21 (FGF21), angiopoietin-like proteins (ANGPTLs), leukocyte cell-derived chemotaxin 2 (LECT2), and selenoprotein P (Sep), which have a positive or negative regulatory effect on the pathogenesis of NAFLD by inducing glucose and lipid metabolism, oxidative stress, and systemic inflammation (6).

#### 2.1.1. Fetuin-A may promote NAFLD by inducing IR and inflammation

Fetuin-A is a phosphorylated glycoprotein mainly synthesized by hepatocytes, which was originally discovered as the natural inhibitor of insulin receptor tyrosine kinase. However, it is recently found that fetuin-A is closely associated with IR, inflammation response, and fatty acid metabolism (6). Excessive accumulation of free fatty acids (FFA) in hepatocytes is the main characteristic of NAFLD that could enhance nuclear factor (NF)-κB binding to the fetuin-A promoter, causing an increase in the synthesis and secretion of fetuin-A (7). Indeed, epidemiological studies revealed that NAFLD patients had considerably greater circulating fetuin-A levels than healthy controls, and elevated fetuin-A independently predicted the occurrence of NAFLD (8). It was reported that fetuin-A was involved in the low-grade inflammation response of NAFLD by acting as an endogenous ligand of toll-like receptor 4 (TLR4) and promoting the secretion of proinflammatory cytokines and chemokine in monocytes, macrophages and adipose tissue (9). Furthermore, fetuin-A also suppressed the expression of the insulin-sensitizing protein adiponectin, which further aggravated IR. In addition, fetuin-A could inhibit transforming growth factor β1 (TGF-β1) and collagen type I α 1 (Col1α1) expression and promote bone morphogenic protein and activin membrane-bound inhibitor (BAMBI) gene expression in human hepatic stellate cell (HSC), resulting in anti-hepatic fibrosis effects in NAFLD patients (10). Collectively, high fetuin-A levels were more common in NAFLD subjects and were involved in the pathogenesis of NAFLD by inducing IR and provoking an inflammatory response. Considering that fetuin-A acted as a bridge between metabolic disorders and inflammatory processes, it could be a potential therapeutic target for metabolic diseases, and more basic experiments and clinical research are needed to confirm these findings.

#### 2.1.2. FGF21 may ameliorate NAFLD by regulating lipid and glucose homeostasis and fatty acid oxidation

FGF21 is a member of the fibroblast growth factor family prominently secreted by the liver, adipose tissues, pancreas, and testes, and its expression is mediated by activating peroxisome proliferator-activated receptor-α (PPAR-α) during fasting or starvation and induced by PPAR-γ after feeding (11). Mounting evidence suggested that serum FGF21 levels were considerably greater in patients with NAFLD in comparison with the controls, and its levels were positively correlated with triglyceride and fasting insulin concentrations, and negatively associated with high-density lipoprotein cholesterol (HDL) and body mass index (BMI) (12). A number of studies revealed that FGF21 also had properties that promote glucose uptake in adipocytes, increase insulin secretion, improve insulin sensitivity, enhance fatty acid oxidation, and attenuate hepatic steatosis (13). For instance, Gong et al. proposed that FGF21 improved hepatic insulin sensitivity and regulated glucose homeostasis by inhibiting mammalian target of rapamycin complex 1 (mTORC1) activation to enhance protein kinase B (Akt) phosphorylation (14). Moreover, circulating FGF21 attenuated high-fat diet-mediated NAFLD by inducing lipophagy via adenosine monophosphate-activated protein kinase (AMPK) dependent pathway (15). Interestingly, two prospective studies in China showed that high serum FGF21 level was an independent predictor of NAFLD and SS, respectively (16, 17). Based on the above findings, it may be reasonable to speculate that FGF21 was a potential biomarker or therapeutic target of NAFLD. However, pre-clinical studies uncovered that the efficacy of FGF21 analogues and mimetics in NASH patients was not satisfactory, suggesting that more effective measures were needed to improve the safety and efficacy of these analogs and mimetics (18). Taken together, FGF21 ameliorated the progression of NAFLD by regulating a series of metabolic processes, such as lipid and glucose homeostasis, fatty acid oxidation, oxidation stress and low-grade inflammation. AMPK signaling may be a key molecular pathway in which FGF21 is closely related to NAFLD pathogenesis.

#### 2.1.3. ANGPTLs may promote NAFLD by affecting lipid metabolism and inflammatory response

ANGPTLs are a family of secretory glycoproteins that share structural similarities to the angiopoietins, which are abundantly expressed in liver and adipose tissue. So far, eight ANGPTLs have been discovered, from ANGPTL1 to ANGPTL8, which are involved in various biological and pathophysiological progress, including glucose and lipid metabolism, inflammation, angiogenesis and atherosclerosis (19). ANGPTL3, ANGPTL4 and ANGPTL8 are the most extensively studied members of ANGPTLs, and a recent meta-analysis included 13 studies (854 NAFLD patients and 610 controls) found that NAFLD patients had significantly higher circulating ANGPTL8 levels than the healthy controls, while the levels of ANGPTL3 and ANGPTL4 were similar between NAFLD patients and control subjects (20). A large body of literature proposed that ANGPTL3, ANGPTL4, and ANGPTL8 played pivotal roles in the pathogenesis of NAFLD by modulating vitamin D receptor (VDR), IR and lipid metabolism process or its key enzymes, especially lipoprotein lipase (LPL) (21). ANGPTLs were novel transcription targets of VDR and activated VDR upregulated hepatic and systemic ANGPTLs expression in NAFLD patients, thus increasing lipid synthesis and intrahepatic fat accumulation and aggravating liver fibrosis (21). A previous study pointed out that ANGPT3 induced adipose tissue lipolysis and increased the release of FFA and glycerol from adipocytes, contributing to IR and liver fat accumulation (22). Likewise, ANGPTL4 also stimulated adipose tissue lipolysis and suppressed the clearance of triglycerides by reducing LPL activity (6). Furthermore, ANGPTL4 improved glucose tolerance and IR by downregulating insulin signaling pathway-associated genes, such as Akt, Janus kinase 2/signal transducer and activator of transcription-3 (JAK2/STAT3), but promoted diet-induced hepatic steatosis (23). ANGPTL8 enhanced insulin sensitivity by directly activating Akt phosphorylation in the insulin-mediated phosphatidylinositol 3 kinase (PI3K)/Akt signaling pathway (24). ANGPTL8 also promoted lipogenesis via upregulating sterol regulatory element-binding protein-1 (SREBP1) (25). Strikingly, ANGPTL8 had been found to negatively regulate NF-κB, a key driver of inflammatory signaling cascades, by modulating the autophagic degradation of inhibitor of κB kinase γ (IKKγ) (26). Although the N-terminal domains of ANGPTL3 and ANGPTL4 influenced lipoprotein metabolism by inhibiting LPL activity, a large community-based study demonstrated that the relationship between plasma ANGPTL3 and ANGPTL4 levels and circulating lipids concentrations was nearly opposite (27). It was worth mentioning that ANGPTL8 did not seem to act LPL alone, rather forming a complex with ANGPTL3 or ANGPTL4 to enhance or weaken the inhibitory effect on LPL activity, respectively. Overall, these findings suggested that ANGPTLs played a certain role in the development of hepatic steatosis by regulating lipid metabolism and inflammatory response. JAK2/STAT3 and PI3K/Akt signaling may be important molecular pathways involved in ANGPTLs and NAFLD development.

#### 2.1.4. LECT2 may promote NAFLD by disturbing IR, inflammation and lipid metabolism

LECT2, a recently discovered hepatocyte-secreted protein, is primarily expressed in the adult and fetal liver. Circulating LECT2 levels are highly sensitive to the change in fat content, and positively correlated with the severity of obesity, NAFLD, IR, hepatic steatosis and inflammation (28, 29). Moreover, serum and hepatic LECT2 expression are significantly increased in NAFLD patients and mice, and bioinformatical analysis uncovered that LECT2 promoted the occurrence and development of NAFLD through the signal transducers and activators of transcription-1 (STAT-1) pathway (30). Interestingly, LECT2-deficient mice exhibited increased insulin sensitivity in skeletal muscle rather than in liver or adipose tissue, but muscle insulin sensitivity of LECT2-deficient mice was attenuated by a high-fat diet, indicating that LECT2 may be a therapeutic target for obesity-induced IR (28). A recent study suggested that LECT2 KO mice had lower M1-like macrophages and M1/M2 ratio that were associated with reduced liver inflammation, implying that higher LECT2 levels acted as a potential predictor of NAFLD progression (29). Additionally, LECT2 was also identified as a functional ligand of Tie1 that exerted a crucial role in hepatic fibrogenesis by activating PPAR signaling and inhibiting endothelial cells migration and tube formations (31). All in all, LECT2 may mediate the progression of NAFLD by promoting liver lipid accumulation and inflammation as well as IR through STAT-1 signaling pathway, which might provide new insights into the diagnosis and treatment of NAFLD.

#### 2.1.5. Sep may promote NAFLD by inducing lipid metabolism disorders

Sep is a glycoprotein mainly synthesized and secreted by the liver and adipose tissue, which belongs to the family of selenium transporter proteins and has the function of transporting selenium (Se) from the liver to extrahepatic organs. Despite clinical and epidemiological data on Sep levels in subjects with NAFLD being limited and contradictory, most studies revealed that NAFLD patients had markedly higher serum Sep levels. With elevated Sep levels, the prevalence rate of NAFLD also increased. Individuals with the highest Sep tertile had a 7.48 times higher risk of NAFLD than those with the lowest Sep tertile, even after adjustments for multiple possible confounding factors (32). Moreover, serum Sep concentrations were negatively correlated with liver attenuation index (a quantitative index of hepatic fat accumulation), assuming that Sep was a novel biomarker of NAFLD (32). Both *in vivo* and *in vitro* studies demonstrated that Sep may exacerbate the development of NAFLD by activating AMPK/ACC (acetyl-CoA carboxylase) pathway (33). However, Se had a beneficial effect on hepatic steatosis, inflammation and fibrosis by enhancing glutathione peroxidase (GSH-Px) activity and inducing HSC apoptosis (34). In aggregate, the above findings indicated that Sep can be considered as the target for the treatment of NAFLD. It is noteworthy that current data on the relationship between Sep or Se and NAFLD were inconsistent, and more extensive and explicit studies are required to elucidate the association.

### 2.2. Adipokines

Adipokines are polypeptides widely present in the adipose tissue, which usually regulate liver fat accumulation, IR, and inflammatory response through autocrine, paracrine and endocrine ways, exerting an important role in the pathogenesis of NAFLD (35). Nowadays, the association between NAFLD and circulating adiponectin and leptin levels has been well-studied, and circulating leptin levels increase, but adiponectin levels decrease with increasing NAFLD severity (35). Notably, the data on the contribution of other adipokines [resistin, retinol binding protein-4 (RBP4), visfatin, chemerin, and irisin] to the occurrence and progression of NAFLD are relatively limited and inconclusive, which needed to be further explored.

#### 2.2.1. Leptin may have a dual role in NAFLD pathogenesis by regulating lipid and glucose metabolism

Leptin is the first discovered adipokine, which modulates metabolism, energy homeostasis and neuroendocrine function mainly through activating the isoform b of leptin receptor (LepRb) and downstream signaling pathways, such as JAK2/STAT3, mitogen-activated protein kinase (MAPK)/extracellular signal-regulated kinase (ERK), and STAT5 pathways (36). Polyzos et al. performed a meta-analysis including 33 studies and 2,612 subjects found that increased leptin levels were indeed more prevalent in patients with NAFLD than the controls, and higher circulating leptin levels were correlated with increased severity of NAFLD (37). It has been accepted that leptin plays a dual action in the pathogenesis of NAFLD. It has effects on increased insulin sensitivity and anti-steatosis by inhibiting insulin secretion and suppressing hepatic glucose production and lipogenesis in the initial stage of the disease (35, 38). When the disease persists or progresses, patients have hyperleptinemia and leptin resistance, which can reduce insulin secretion and lead to hyperinsulinemia, resulting in IR and further exacerbation of disease (38). Therefore, timely intervention to reduce circulating leptin levels is essential to manage the development of NAFLD. However, the aforementioned dual leptin roles have not yet been confirmed in the NAFLD population, and the exact mechanism remains unclear, so further large prospective research should be performed to validate these findings.

#### 2.2.2. Adiponectin may alleviate NAFLD by reducing inflammation, IR and lipid disturbances

Adiponectin is one of the abundant and adipocyte complement-related adipokines that is predominantly produced by adipocytes and hepatocytes. Contrary to other adipokines, the levels of adiponectin decrease with the increase of adipose mass. A meta-analysis of 27 studies and 2,243 individuals (1,545 NAFLD patients and 698 controls) indicated that patients with NAFLD had considerably lower adiponectin levels compared with the controls [weighted mean difference (WMD) = 4.16, 95% CI: 2.63–5.69]. Meanwhile, lower levels of adiponectin were also observed in SS patients than that in NASH patients (WMD = 1.81, 95% CI: 1.09–2.53) (39). Adiponectin acted mainly through two transmembrane receptors (AdipoR1 and AdipoR2), mostly expressed in skeletal muscle and moderately presented in the liver. Emerging evidence showed that adiponectin participated in the pathogenesis of liver diseases through PPAR-α and AMPK signaling pathways (40). In addition, some studies also demonstrated that adiponectin exerted insulin-sensitizing, anti-steatosis, anti-fibrotic, anti-inflammatory and anti-atherosclerosis effects by decreasing gluconeogenesis and *de novo* lipogenesis, inhibiting ectopic fat deposition, stimulating FFA oxidation, reducing HSC activation and proliferation, and inducing the disorder of anti-inflammatory and pro-inflammatory cytokines (35). These properties made it an attractive therapeutic target in NAFLD individuals. Taken together, NAFLD patients had lower adiponectin levels, and adiponectin had protective effects on the pathogenesis of NAFLD by activating PPAR-α and AMPK signaling pathways through AdipoR1 and AdipoR2.

#### 2.2.3. Resistin may promote NAFLD by inducing inflammation, IR and hepatic steatosis

Resistin is a polypeptide secreted by adipose tissue, hepatocytes and inflammatory cells, especially macrophages, which participates in glucose and lipid metabolism and promotes the development of IR and obesity. The liver is the main target organ of resistin, and resistin production seems to evaluate with the increase of liver injury. A recent meta-analysis revealed that adult NAFLD patients had higher serum resistin levels compared with healthy controls [standardized mean difference (SMD) = 0.522, 95% CI: 0.004–1.040], but there was no obvious difference in serum resistin levels between SS and NASH patients (SMD = 0.15, 95% CI: −0.06 to 0.36) and between SS and controls (SMD = −0.34, 95% CI: −0.91 to 0.23) (41). Accumulating evidence delineated that resistin can not only induce hepatocyte steatosis and IR by modulating hepatic lipid metabolism and increasing liver fat content but also stimulate inflammatory cells and HSC to release proinflammatory cytokines, such as tumor necrosis factor-α (TNF-α), interleukin-6 (IL-6), interleukin-8 (IL-8) and monocyte chemotactic protein 1 (MCP-1), by activating NF-κB signaling pathway (35). In addition, resistin also activated HSC and promoted the production of TGF-β and type I collagen by Kupffer cells, therefore having a pro-fibrotic effect on hepatocytes (42). In summary, increased resistin levels were common in NAFLD patients, and its expression was positively correlated with hepatic steatosis, inflammation, and fibrosis. Based on the above findings, resistin is is considered to be an attractive therapeutic target during disease progression.

#### 2.2.4. Visfatin may promote NAFLD by initiating inflammation and glucose metabolism disorders

Visfatin, known as pre-B cell colony-enhancing factor (PBEF) or nicotinamide phosphoribosyltransferase (NAMPT), could be expressed in various tissues and involved in the pathogenesis of a wide range of diseases, such as CVD, metabolic and inflammatory disorders, cancers and aging. The latest meta-analysis of 21 studies and 1,923 individuals indicated no significant change in serum visfatin levels between NAFLD, SS, NASH patients and control subjects (43). Visfatin had NAMPT activity that could regulate glucose-stimulated insulin secretion in pancreatic β-cell (35). Wang et al. pointed out that inhibition of NAMPT activity aggravated high diet-induced hepatic steatosis in mice by regulating the Sirt1/AMPKα/SREBP1 signaling pathway (44). Visfatin also enhanced the secretion of proinflammatory cytokines in CD14(+) monocytes, such as IL-6, TNF-α, and IL-1β, and inhibited insulin signaling transduction through STAT3 and NF-κB signaling pathways in hepatocytes (45, 46). Nevertheless, there were few studies to explore the role of visfatin in liver fibrosis in detail. Limited evidence demonstrated that visfatin promoted CCL20 expression in macrophages via NF-κB and MAPK kinase (MKK)3/6-p38 signaling pathways and increased the expression of fibrosis markers in HSC (47). On the whole, existing studies do not support the specific role of visfatin in development of NAFLD. Visfatin may have anti-steatotic, proinflammatory, and pro-fibrotic effects, but it remains to be clarified through mechanistic studies.

#### 2.2.5. RBP4 may promote NAFLD by inducing glucose and lipid metabolism disorders

RBP4 is a newly identified adipokine that is mostly expressed and produced in visceral adipose tissue and the liver, and it plays a key role in regulating glucose and lipid metabolism (35). Previous research delineated that RBP4 could increase basal glucose production and hepatic lipogenesis by enhancing the expression of the gluconeogenic enzyme phosphoenolpyruvate carboxykinase (PEPCK) in hepatocytes and regulating SREBP1 and activating PPAR-γ coactivator 1β (PGC-1β), respectively (48, 49). Besides, RBP4 also induced hepatic mitochondrial dysfunction by reducing mitochondrial content and attenuating fatty acid β-oxidation, leading to hepatic steatosis. To date, the relationship between NAFLD and circulating RBP4 levels is still inconclusive. Zhou et al. evaluated 12 studies and 4,247 participants (2,271 NAFLD patients and 1,976 controls) and found that there was an insignificant difference in circulating RBP4 levels in NAFLD patients versus controls (SMD = 0.08, 95% CI: −0.21 to 0.38), NASH patients versus controls (SMD = −0.49, 95% CI: −1.09 to 0.12), SS patients versus controls (SMD = −0.72, 95% CI: −1.64 to 0.20), and NASH patients versus SS patients (SMD = −0.04, 95% CI: −0.32 to 0.24) (50). Therefore, they supposed that circulating RBP4 levels may not be associated with NAFLD. Nonetheless, a prospective longitudinal cohort study with a 3.09-year follow-up reported that serum RBP4 concentrations were positively correlated with incident NAFLD and negatively correlated with NAFLD regression (51). The inconsistency of the above findings may be owing to the differences in study design and NAFLD diagnostic criteria, as well as the heterogeneity of the participants. All in all, the research on the link between RBP4 and NAFLD is still controversial, and it is necessary to conduct more large-scale clinical and basic studies to elucidate whether NAFLD promotes or inhibits RBP4 expression, and whether RBP4 has a distinct role in specific hepatic lesions.

#### 2.2.6. Chemerin may ameliorate NAFLD by reducing inflammation and oxidative stress

Chemerin is mainly secreted by adipocytes and hepatocytes in an inactive form and activated by extracellular C-terminal protease cleavage, which acts as a ligand to bind to chemokine-like receptor 1, G protein-coupled receptors and chemokine (CC motif) receptor-like 2 (52). Chemerin is strongly related to a variety of physiological or pathological activities, such as innate immunity, inflammation, fibrosis, endothelial dysfunction, angiogenesis, oxidative stress, IR, and metabolic disorders (35, 53). Epidemiological studies reported that serum chemerin levels were generally higher in NAFLD or SS patients relative to controls, but there was no evident difference in circulating chemerin levels between NASH and healthy controls, and between NASH and SS patients (53). Of note, although the data on hepatic chemerin mRNA expression in NASH patients were inconsistent, most studies indicated that hepatic chemerin mRNA expression was closely correlated with inflammation and hepatic fibrosis (54). In addition, basic research proposed that chemerin also ameliorated liver steatosis, lobular and portal inflammation in NASH mice by promoting autophagy and alleviating oxidative stress through JAK2/STAT3 phosphorylation (55). To sum up, chemerin acts as an indispensable regulator in the onset of NAFLD, and more detailed mechanistic research are necessary.

#### 2.2.7. Irisin may ameliorate NAFLD by inhibiting lipid accumulation and inflammation

Irisin, an exercise-induced myokine and adipokine, is implicated in the pathogenesis of multiple metabolic diseases, including obesity, diabetes mellitus, dyslipidemia, NAFLD, and CVD (56). More recently, two meta-analyses assessed circulating irisin levels in NAFLD patients, and the results suggested that irisin levels in NAFLD patients were similar to that in control populations and were less likely to be affected by disease severity and racial/ethnic disparities (57, 58). Current evidence based on experimental models proposed that irisin can not only induce the browning of white adipose tissue and increase thermogenesis but also reduce hepatic gluconeogenesis and prevent lipid accumulation in hepatocytes through the AMPK pathway (56). Additionally, irisin inhibited inflammation by competitive binding with myeloid differentiation factor 2 (MD2) to improve NAFLD (59). Yet, there is little population-based research on the association between irisin and NAFLD or specific hepatic lesions. In short, more studies are needed to explore the role of irisin in the occurrence and progression of NAFLD. The protective effect of irisin on NAFLD may be achieved through AMPK signaling pathway.

### 2.3. Cytokines and complements

Growing evidence disclosed that cytokines were the key mediators of signal transmission among live cells, which may be critically participated in the pathophysiology of NAFLD by inducing hepatic inflammation, IR and fibrosis (60). As an essential part of innate immune system, complements and their cleavage products were mostly produced by hepatocytes, which also played a fundamental role in the pathogenesis of NAFLD (61).

#### 2.3.1. Cytokines may promote NAFLD by fueling inflammation, IR and lipid accumulation

Cytokines are pleiotropic regulatory peptides synthesized and secreted by immune cells and non-immune cells, including most types of hepatocytes, consisting mainly of IL, TNF, chemokines, interferon, colony-stimulating factor and lymphokines subfamily. Accumulating research indicated that cytokines were essential for the occurrence and progression of NAFLD through stimulating hepatic inflammation and apoptosis, as well as inducing IR and fibrosis (60). Notably, two key proinflammation cytokines, TNF-α and IL-6, were involved in the initial events of many types of liver injury, and triggered the secretion of other cytokines, contributing to the activation of the inflammatory cascade (62). It was reported that circulatory expression of TNF-α and IL-6 were upregulated in both NAFLD patients and mouse NAFLD models, and were strongly correlated with an increased risk of NAFLD (62). Elevated TNF-α modulated systemic and hepatic insulin sensitivity through multiple mechanisms, including downregulating insulin activation on its receptor, decreasing glucose uptake and transport by inhibiting glucose transport 4 (GLUT4) in adipocytes, reducing insulin sensitivity by suppressing adiponectin activity and attenuating insulin signal transduction by depressing insulin receptor phosphorylation (63). Furthermore, TNF-α could reduce fat decomposition in peripheral tissue adipolysis and promote the synthesis and accumulation of triglycerides in hepatocytes. Indeed, TNF-α polymorphisms were also closely associated with susceptibility to NAFLD. The role of IL-6 in the development of NAFLD was complicated, which was initially employed as a hepatoprotector in hepatic steatosis via reducing oxidative stress and preventing mitochondrial dysfunction (64). Nonetheless, IL-6 could also sensitize the liver to injury, inhibit LPL activity, promote hepatocyte apoptosis and induce IR, thus, it was like to play a deleterious role in NAFLD pathogenesis (60). In addition, accumulating evidence indicated that growth differentiation factor 15 (GDF15) also played an indispensable role in the pathogenesis of NAFLD (65–67). *In vivo* and *in vitro* NASH models, GDF15 expression was upregulated in the hepatocytes, and its concentration was also increased with the progression of the disease, especially in subjects with advanced fibrosis (67). Among patients with NAFLD, the highest quartile of circulating GDF15 was positively associated with the risk of advanced fibrosis after adjustment for age, sex, BMI, and other factors (68). Furthermore, recombinant GDF15 or GDF15 overexpression ameliorated NASH and related metabolic disorders in mice by alleviating hepatic inflammatory response and modulating lipid metabolism and β-oxidation of fatty acids, which was independently of reduction in body mass (66, 67). Moreover, the phosphorylation of SMAD2 and SMAD3 likely exert a certain role in GDF15-induced fibrogenesis (68). These findings revealed that elevated GFD15 was an independent risk factor for advanced fibrosis in NAFLD subjects, and it could be a promising therapeutic target for NAFLD and NASH. Collectively, high TNF-α levels contribute to an increased risk of NAFLD through modulating inflammation, lipid accumulation and insulin sensitivity, and elevated GFD15 might exert positive effects in NASH and NAFLD subjects. However, the exact role of IL-6 in the progression of NAFLD remains unclear and further research is needed.

Chemokines are a class of highly conserved small cytokines primarily produced by hepatocytes, HSC, Kupffer cells, endothelial cells and circulating immune cells. A growing number of studies demonstrated that chemokines and their receptors levels were elevated in NAFLD subjects and acted indispensable roles in mediating IR, inflammation, hepatic steatosis and fibrosis, especially chemokine ligand 2 (CCL2), CCL5, and CCL20 (60, 69). CCL2, also named MCP-1, usually activates target cells by binding to its receptor CCR2, and its levels were positively related to the hepatic fat content in NAFLD patients (60). Accumulating data revealed that CCL2 not only directly contributed to lipid accumulation in hepatocytes via activating PPAR-α but also promoted liver inflammation by inducing the recruitment of CCR2 expression in bone marrow-derived monocytes (60, 69). In addition, CCL2 and CCR2 also exhibited a certain role in HSC recruitment and activation that aggravated liver fibrosis. Similarly, there was a well-established link between CCL5-CCR5 and hepatic steatosis, inflammation, fibrosis and IR via monocyte/macrophage and HSC recruitment and activation (69). Hanson et al. found that CCR20 expression increased with NAFDL severity and HSC activation, which was regulated by miR-590-5p (70). All of the above evidence suggests that cytokines and chemokines are involved in the development and progression of NAFLD by inducing inflammation, hepatic lipid accumulation, and insulin sensitivity, but whether they could be used as therapeutic or diagnostic targets for NAFLD still needs to be confirmed.

#### 2.3.2. Complements may promote NAFLD by affecting inflammation and lipid metabolism

The complement system is an important part of immunity and inflammation that may be involved in the pathogenesis of NAFLD by modulating IR, hepatic inflammation, and lipid metabolism disorders (61). Under physiological conditions, a potential balance exists between complement system activation and hepatocyte lipid metabolism, which helps to maintain liver homeostasis. Nevertheless, this balance is disrupted during the progression of NAFLD, leading to the disturbance of lipid metabolism in hepatocytes and the aggravation of hepatic steatosis (71). Epidemiological data proposed that circulating complements and their cleavage product concentrations (such as C3, C3a, and C5a) were increased in NAFLD and other metabolic disorders. It was worth mentioning that complement C3 played a pivotal role in the cascade-activated complement system, and the accumulation of C3 activation products around hepatocytes further aggravated the progression of NAFLD (72). Moreover, serum C3 levels were considered as an independent risk factor for NAFLD diagnosis and positively correlated with the prevalence and severity of NAFLD (71). In addition, recent research elucidated that C3a and C5a had chemotactic and pro-inflammatory properties associated with NAFLD (61). Together, these findings can provide some evidence for complements as novel therapeutic strategies.

### 2.4. Extracellular vesicles

Extracellular vesicles (EVs) are nano-sized vesicles surrounded by a lipid bilayer that can be released by most cells. EVs, mainly including small EVs (sEVs) and microvesicles, have the function of mediating intercellular communication within tissues and organs through transferring proteins, lipids, and RNAs. Excessive lipid overload and accumulation were major characteristics of NAFLD that can trigger hepatocytes to produce and release EVs, and the latter also participated in the initiation and progression of hepatic inflammation, steatosis, fibrosis, and HCC (73, 74). Accumulating data demonstrated that hepatocytes-derived EVs not only activate macrophage-associated inflammation response via TNF-related apoptosis-inducing ligand (TRAIL) in a death receptor (DR5)-dependent manner but also promote endothelial inflammation and atherogenesis via microRNA-1 (75). Hepatocyte-derived sEVs also promoted hepatic fibrosis in NAFLD by directly modulating HSC phenotype and up-regulating the expression of profibrogenic genes, including Col1α1, α-smooth muscle actin (αSMA), and tissue inhibitor of metalloproteinase-2 (TIMP-2) (76). Furthermore, HSC-derived sEVs contained connective tissue growth factor (CCN2), and its concentration increased with HSC activation, which may amplify or fine-tune fibrotic signal transduction and may be considered as a potential candidate biomarker to assess hepatic fibrosis (77). Besides, HSC-derived EVs also contained C-X-C motif ligand 10 (CXCL10), hexokinase 1 (HK1), vascular endothelial growth factor (VEGF), matrix metalloprotein 2 (MMP2) and MMP9, which facilitated tumor growth and transfer, as well as angiogenesis (78). Notably, messenger RNA (mRNA), microRNA (miRNA) and long non-coding RNA (lncRNA) have been found in EVs that elicit a pivotal role in the pathogenesis of NAFLD, especially miR-122, miR-192, miR-21 and miR-7 (79). miR-122 and miR-192 were the most abundant RNAs in hepatocytes, and hepatic miR-122 levels were upregulated during early NAFLD development but decreased gradually with progression towards NASH and hepatic fibrosis (79, 80). Previous studies revealed that miR-122 knockdown in diet-induced NAFLD mouse model could inhibit hepatic lipogenesis via liver kinase B1 (LKB1)/AMPK signaling pathway by targeting Sirt1 (81). Moreover, miR-122 had the capacity to protect against hepatic inflammation and fibrosis by targeting Relb, FN1 and SRF, respectively (79). Therefore, miR-122 may be a potential diagnostic and/or prognostic marker of NAFLD patients. Numerous studies presented that circulating miR-122 had good accuracy in NAFLD diagnosis, with the area under the receiver operating curve of 0.67–0.93 (76). However, miR-192 and miR-21 expression increased with NAFLD progression, and miR-192 had pro-fibrotic properties by enhancing Col1α1, αSMA and TGF-β1 expression in HSC (76). miR-21 induced hepatic steatosis by increasing lipogenesis and promoted hepatic inflammation and fibrosis by inhibiting PPAR-α (82). Similarly, miR-7 levels were significantly elevated in NAFLD mouse model, triggering NLRP3 (NOD-like receptor protein 3) inflammasome activation and coronary microvascular endothelial hyperpermeability through regulation of lysosomal associated membrane protein 1 (LAMP1) and Cathepsin B (83). Therefore, miR-7 acted as a bridge between NAFLD and coronary diseases by targeting the LAMP1/Cathepsin B/NLRP3 signaling pathway. On the whole, EVs are also involved in the pathogenesis of NAFLD and can be acted as novel diagnostic biomarkers and therapeutic targets.

### 2.5. Gut-derived factors

The liver interacts with the gut through the portal vein, and the liver is able to remove a large number of bacterial products, toxins and metabolites from the gut. However, NAFLD patients have excessive bacterial overgrowth, intestinal dysbiosis and metabolic product alterations, as well as increased intestinal permeability, which facilitates the translocation of pathogen-associated molecular pattern (PAMP)/microbial-associated molecular pattern (MAMP) to the liver, inducing hepatic and systemic inflammation and immune responses (84, 85). In particular, increasing research focus on the association between NAFLD and microbial metabolites, such as trimethylamine N-oxide (TMAO), lipopolysaccharide (LPS), bile acids, short-chain fatty acids (SCFAs), and N,N,N-trimethyl-5-aminovaleric acid (TMAVA) (86–89). Recent meta-analyses showed that NAFLD patients had significantly higher concentrations of circulating TMAO (SMD = 0.66, 95% CI: −0.12 to 1.21), and plasma TMAO levels were related to all-cause mortality in NAFLD subjects, independently of traditional risk factors (90, 91). TMAO participates in the initiation of NAFLD through a variety of mechanisms. On the one hand, TMAO promotes IR by regulating glucose metabolism and increasing the levels of CCL2 and inflammatory cytokine C-C motif chemokine ligand 2; on the other hand, it exacerbates hepatic steatosis by reducing the conversion of cholesterol into bile acids and inhibiting farnesoid X receptor (FXR) signaling (86). However, TMAO attenuates liver fibrosis by inhibiting ATP1B1 expression in vascular endothelial cells (88). LPS, also named as endotoxin, was identified as a diagnostic biomarker for NAFLD, which can interact with its receptor TLR4 in inflammatory cells (e.g., macrophages and platelets) to initiate inflammatory processes and augment NAFLD susceptibility (92). Higher endotoxin levels are indeed more prevalent in NAFLD patients versus liver-healthy controls, and its levels are associated with circulating C-reactive protein (CRP) concentrations (93). However, intestine-derived HDL3 can restrain liver injure by preventing LPS binding to hepatic macrophages and inflammatory activation through the portal vein (94). Bile acids are crucial regulators of insulin sensitivity, glucolipid metabolism and immune response, which contribute to the pathogenesis of NAFLD by regulating FXR and Takeda G-protein receptor 5 (TGR5) (3). FXR activation alleviates the progression of NAFLD through reduced lipogenesis and lipid absorption mediated by bile acids, increased FFA oxidation, affected cholesterol transportation, and inhibited NF-κB-mediated inflammatory signaling pathways (92). TGR5 activation not only modulates liver inflammation through TGR5-cAMP dependent pathways, but also induces the release of glucagon-like peptide-1 (GLP-1) and represses the activation of NLRP3 inflammasome to regulate glucose homeostasis (86). SCFAs, mainly include acetate, propionate, and butyrate, which activates the G-protein-coupled receptors (GPCRs) GPR41 and GPR43, and the latter stimulates the secretion of GLP-1 and peptide-YY in the gut enteroendocrine L cells, therefore enhancing lipid and FFA oxidation, increasing insulin sensitivity, as well as promoting hepatic lipogenesis through AMPK-dependent pathway (86, 95). In addition, SCFAs also reduces hepatic inflammation and steatosis by increasing c-Jun N-terminal kinase (JNK) and NF-κB phosphorylation, inhibiting the secretion of proinflammatory cytokines, and suppressing the activity of histone acetyltransferases to reduce the generation of regulatory T cells (96). Zhao et al. indicated that plasma TMAVA levels were increased in patients with liver steatosis compared with healthy controls, and TMAVA treatment exacerbated hepatic steatosis and caused gut microbial imbalance in mice (89). Furthermore, intestinal microbes metabolized trimethyllysine to TMAVA, which inhibited carnitine biosynthesis by competitively the binding of γ-BB to γ-butyrobetaine hydroxylase (BBOX) and reduced hepatic fatty acid oxidation (FAO), thereby promoting hepatic steatosis (89). Besides, TMAVA was a key determinant for the pathogenesis of cardiac hypertrophy through disturbed cardiac energy metabolism and altered mitochondrial ultrastructure, resulting in inhibition of FAO, myocardial lipid accumulation and carnitine synthesis (97). In summary, gut-derived factors are believed to play an indispensable role in the onset of NAFLD. TMAO has the properties of aggravating hepatic steatosis and alleviating liver fibrosis. SCFAs have beneficial roles in reducing liver inflammation and steatosis. LPS-TLR4, FXR, TGR5-cAMP, AMPK signaling may be the promising pathways to regulate the progression of NAFLD. TMAVA exacerbates liver steatosis and cardiac hypertrophy by inhibiting carnitine synthesis and hepatic fatty acid oxidation.

## 3. Metabolic organ-secreted factors linking NAFLD to CVD

**Figure 2:**
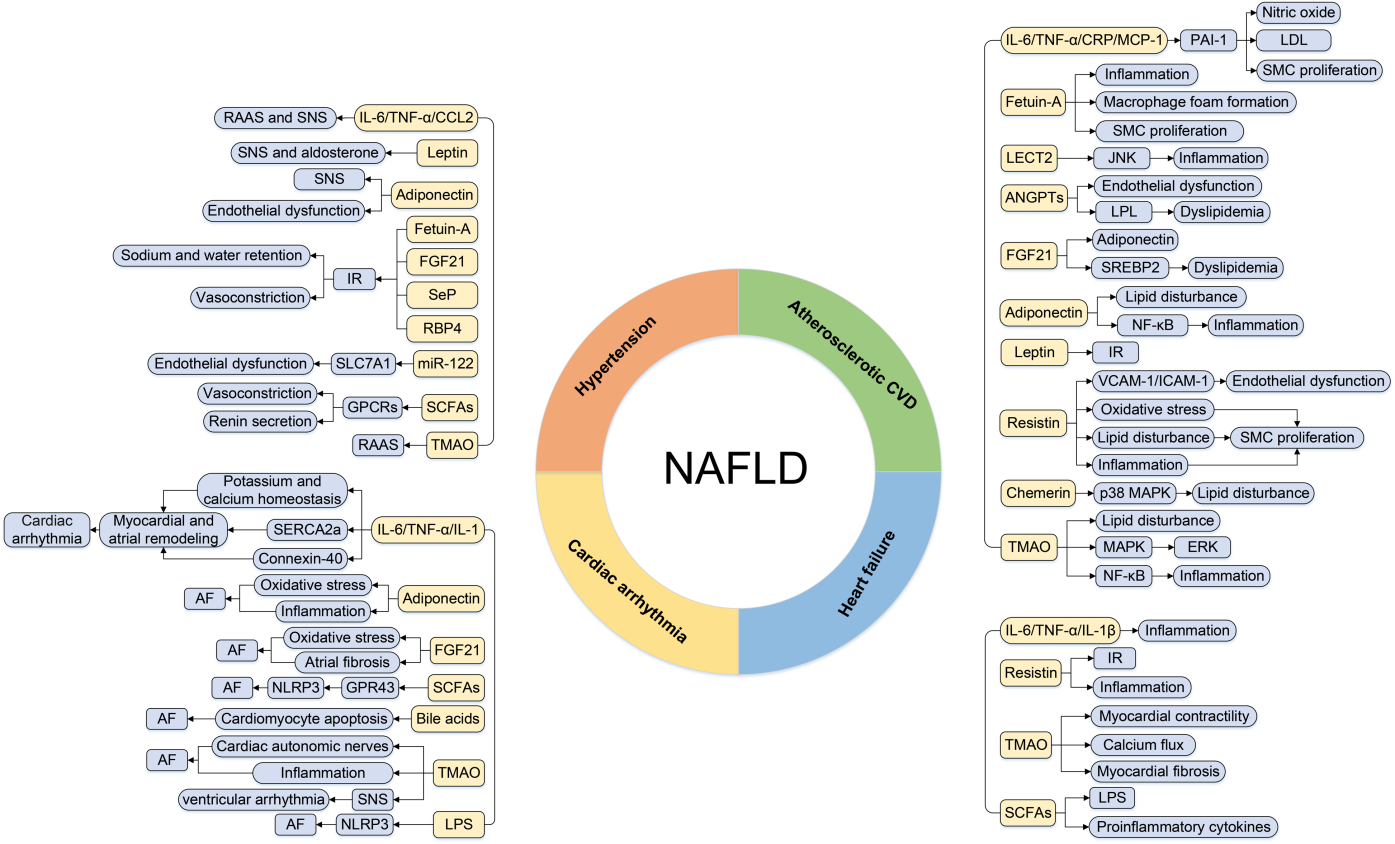
The regulatory roles of metabolic organ-secreted factors linking NAFLD and CVD. AF, atrial fibrillation; ANGPTLs, angiopoietin-like proteins; CCL2, chemokine ligand 2; CRP, C-reactive protein; CVD, cardiovascular disease; ERK, extracellular signal-regulated kinase; FGF21, fibroblast growth factor 21; JNK, c-Jun N-terminal kinase; GPCRs, G-protein-coupled receptors; ICAM-1, intercellular adhesion molecule-1; IL-1, interleukin-1; IL-1β, interleukin-1β; IL-6, interleukin-6; IR, insulin resistance; LDL, low-density lipoprotein; LECT2, leukocyte cell-derived chemotaxin 2; LPL, lipoprotein lipase; LPS, lipopolysaccharide; MAPK, mitogen-activated protein kinase; MCP-1, monocyte chemotactic protein 1; NAFLD, nonalcoholic fatty liver disease; NLRP3, NOD-like receptor protein 3; PAI-1, plasminogen activator inhibitor-1; RAAS, renin-angiotensin system; RBP4, retinol binding protein-4; SCFAs, short-chain fatty acids; Sep, selenoprotein P; SERCA2a, sarco-endoplasmic reticulum Ca2+ ATPase 2a; SMC, smooth muscle cell; SNS, sympathetic nervous system; SREBP2, sterol regulatory element-binding protein-2; TGF-α, transforming growth factor-α; TMAO, trimethylamine N-oxide; VCAM-1, vascular cell adhesion molecule-1.

As well as other metabolic diseases, cardiac involvement in NAFLD subjects is primarily manifested as hypertension, atherosclerotic disease, cardiac arrhythmia, and heart failure (HF). Emerging evidence indicated that the above metabolic organ-secreted factors were linked with the pathogenesis of CVD in NAFLD patients (3). The detailed associations between metabolic organ-secreted factors and CVD in NAFLD patients are described below (Figure 2).

### 3.1. Hypertension

A bidirectional association between hypertension and NAFLD has been proposed, and recent systemic reviews and meta-analyses have indicated that NAFLD is significantly associated with an increased risk of developing hypertension (98). Several prospective studies disclosed that the presence of NAFLD independently predicted the risk of incident hypertension, and the incidence rate of hypertension increased with the severity of NAFLD (99). A large amount of evidence delineated that systemic inflammation, IR, renin-angiotensin system (RAAS) and sympathetic nervous system (SNS) activation may be the potential pathophysiological mechanisms of hypertension in NAFLD patients. Systemic inflammation induced by NAFLD can not only activate RAAS and SNS by elevated circulating cytokines and complements levels (e.g., IL-6, TNF-α, and CCL2) but also alter adipokine profiles characterized by increased leptin levels and reduced adiponectin concentrations (99). Moreover, IL-6 and TNF-α also promote the production of RAAS components, especially angiotensinogen, further contributing to systemic and local angiotensin II formation and hypertension development (100). In addition, higher leptin levels are involved in the pathogenesis of hypertension by sex-specific mechanisms that increase sympathetic activation in males and aldosterone production in females, respectively, while lower adiponectin concentrations regulate hypertension development by increasing SNS activity and induing endothelial dysfunction (101). On the other hand, other altered hepatokine and adipokine profiles in NAFLD subjects, including fetuin-A, SeP, RBP4, and FGF21, have been increasingly recognized to induce systemic IR by impairing insulin signaling, activating proinflammatory signaling and affecting lipid and glucose metabolism disorders. Hyperinsulinemia and hyperglycemia caused by IR are strongly associated with sodium and water retention and vasoconstriction, which play a vital role in the development of hypertension. Until now, very few studies have explored the relationship between EVs, gut-derived factors and hypertension in NAFLD patients. Limited evidence presented that miR-122 may cause the depression of 3′-untranslated region (UTR) variant in SLC7A1 gene expression, further leading to endothelial dysfunction and increased susceptibility to hypertension (102). SCFAs had hypotensive properties by inducing arterial vasodilation and renin secretion via GPCRs pathways, and regulating anti-inflammatory immune cells, especially regulatory T cells (103). On the contrary, TMAO had a detrimental impact on blood pressure via promoting inflammatory responses and activating RAAS, and there was a positive dose-dependent association between plasma TMAO levels and the prevalence of hypertension (104, 105). Together, substantial evidence indicated that altered hepatokine (fetuin-A, SeP, and FGF21), adipokine (leptin, adiponectin, and RBP4), cytokines (IL-6 and TNF-α), EVs (miR-122), and gut-derived factors (SCFAs and TMAO) in NAFLD patients participated in the development of hypertension through inducing inflammation, IR, endothelial dysfunction, and RAAS and SNS activation. However, whether these metabolic organ-secreted factors can be used as diagnostic and prognostic markers or therapeutic targets for hypertension in NAFLD subjects is not yet known and remains to be further explained.

### 3.2. Atherosclerotic CVD

Multiple levels of evidence indicated that subclinical atherosclerosis and atherosclerotic CVD were more prevalent in NAFLD patients, and the prevalence of carotid atherosclerosis and coronary heart disease (CHD) in NAFLD subjects were found to be 35.02% and 44.6%, respectively (106, 107). Meanwhile, individuals with NAFLD had a significantly higher risk of carotid atherosclerosis (OR = 3.2, 95% CI: 2.37–4.32), CHD (OR = 1.33, 95% CI: 1.21–1.45) and acute myocardial infarction (HR = 1.17, 95% CI:1.05–1.30) than non-NAFLD controls, and NAFLD had been proved to be an independent risk factor for atherosclerotic CVD (106–108). Several studies reported that NAFLD was also strongly associated with the markers of subclinical atherosclerosis, such as carotid intima-media thickness (CIMT), carotid plaque, arterial stiffness and coronary artery calcium (CAC) (107, 109). Although NAFLD can promote and aggravate atherosclerosis development, the exact pathogenesis is still poorly unclear. The potential mechanisms for accelerating atherosclerotic CVD in NAFLD patients were complex and might be related to lipid disturbances, IR, chronic inflammation, and endothelial dysfunction. Patients with NAFLD had a pro-atherosclerotic lipid profile characterized by high triglyceride, elevated very low-density lipoprotein (VLDL), high apolipoprotein B (ApoB) to apolipoprotein AI (ApoAI) ratio, and low levels of HDL, which lead to an increased CVD risk (110). IR was a pivotal characteristic of NAFLD pathogenesis that contributed to increased FFA concentrations, caused lipotoxicity, damaged endothelium-dependent vasodilation, enhanced oxidative stress, and had cardiotoxic effects. In addition, in subjects with NAFLD, inflammation appeared as increased levels of cytokines and chemokines in peripheral blood, such as IL-6, TNF-α, CRP, and MCP-1, which further accelerated atherosclerotic CVD by increasing plasminogen activator inhibitor-1 (PAI-1) and adhesion molecules levels in endothelial cells, reducing nitric oxide production, promoting the influx of low-density lipoprotein (LDL) into macrophages, and stimulating vascular smooth muscle cell proliferation (110). Besides, endothelial dysfunction and impaired endothelial flow-mediated vasodilatation observed in NAFLD patients could cause chronic inflammation, increased vasoconstriction, and augmented production of prothrombotic factors, resulting in an increased risk of atherosclerotic cardiovascular events (111). Furthermore, endothelial dysfunction was regarded as an independent risk factor for the occurrence and development of CHD (111). In short, lipid disturbances, IR, inflammation, and endothelial dysfunction are strongly associated with a higher risk of atherosclerotic CVD in NAFLD patients.

Recent studies focused on the potential role of hepatokines (fetuin-A, FGF21, LECT2, and ANGPTLs), adipokines (adiponectin, leptin, resistin and chemerin) and gut-derived factors (TMAO, bile acids and SCFAs) in the development of atherosclerotic CVD in NAFLD patients. These hepatokines and adipokines not only directly act on vascular cells but also regulate other metabolic components, such as IR, systemic inflammation and dyslipidemia, to involve in the pathogenesis of CVD. Among them, elevated fetuin-A levels in NAFLD promote an inflammatory response in vascular endothelial cells, macrophage foam formation, smooth muscle cell proliferation and collagen deposition, contributing to an increased risk of atherosclerotic disease (112). LECT2 may enhance atherosclerotic inflammatory response in endothelial cells through CD209 receptor-mediated JNK phosphorylation (113). ANGPT3, ANGPTL4 and ANGPTL8 can induce endothelial dysfunction and promote dyslipidemia by suppressing the LPL activity, which is associated with a higher risk of atherosclerosis (114). Contrary to other hepatokines, FGF21 has anti-atherosclerotic properties by inducing adiponectin production in adipocytes and by reducing cholesterol biosynthesis through inhibiting hepatic SREBP2 expression (115). Adiponectin has insulin-sensitizing and anti-inflammation effects, which can stimulate vascular endothelial nitric oxide synthase expression, improve atherogenic lipid profile, selectively inhibits endothelial cell apoptosis, and regulate the NF-κB signaling pathway, thereby slowing the progression of atherosclerosis and cardiovascular events (116). Additionally, elevated leptin, resistin, and chemerin concentrations are common in NAFLD patients that also play a crucial role in the aggravation of NAFLD and the development of coronary atherosclerosis, and the potential mechanisms are described below (116, 117). Hyperleptinemia results in selective leptin resistance that has a synergistic effect with IR, which can reduce adiponectin levels and nitric oxide synthase, promote increased IMT, and is considered a predictor of CVD (116). Resistin can induce vascular endothelial dysfunction by triggering vascular cell adhesion molecule-1 (VCAM-1) and intercellular adhesion molecule-1 (ICAM-1) release, promote the proliferation and migration of vascular smooth muscle cells by activating various intracellular signal transduction and enhance oxidative stress, vascular inflammation and lipid accumulation (116, 117). Chemerin increases atherosclerotic CVD risk by affecting the adhesion and migration of human endothelial progenitor cells and exacerbating lipid accumulation in atherosclerotic plaques through p38 MAPK signaling pathway (118). TMAO is considered as a proatherogenic metabolite, which is involved in atherosclerotic CVD by inhibiting reverse cholesterol transport, facilitating migration of macrophage foam cells to the vascular wall, promoting platelet reactivity and thrombosis, and exacerbating inflammatory gene expression through activation of MAPK/ERK and NF-κB pathways (103, 119). Bile acids mainly modulate inflammation and lipid and glucose metabolism through liver FXR and G protein-coupled bile acid receptor 1 (GPBAR1), especially VLDL and triglyceride production, *de novo* fatty acid synthesis and IR, ultimately regulating the progression of CVD in NAFLD patients (120). Notably, SCFAs play the protective effects on atherosclerotic CVD by decreasing pro-inflammatory cytokines (IL-6, IL-8) and VCAM-1 expression, suppressing histone deacetylases and activation of NF-κB inflammatory pathways, inhibiting endothelial ROS formation, and improving pro-atherogenic plasma lipid profile (121). Collectively, the development of subclinical atherosclerosis and atherosclerotic CVD in NAFLD patients may also be partly attributable to metabolic organ-secreted factors. In addition to the above-mentioned factors, the role of other metabolic organ-secreted factors in the pathogenesis of atherosclerotic CVD in NAFLD patients still needs further exploration.

### 3.3. Cardiac arrhythmia

In recent years, a number of studies focused on the relationship between NAFLD and cardiac arrhythmia, especially atrial fibrillation (AF). Several systemic reviews and meta-analyses suggested that NAFLD was associated with high risk of AF (OR = 1.27, 95% CI: 1.18–1.37), prolonged QT interval (OR = 2.86, 95% CI: 1.64–4.99), heart block (HR = 2.65, 95% CI: 1.88–3.72), premature atrial/ventricular contraction (OR = 2.53, 95% CI: 1.70–3.78), and cardiac conduction defect (OR = 5.17, 95% CI: 1.34–20.01) (122–124). Accumulated studies have demonstrated that NAFLD is often accompanied by impaired lipid and glucose metabolism, IR, oxidative stress, systemic inflammation and epicardial adipose tissue (EAT) accumulation, resulting in structural, functional, and electrical remodeling of the heart and predisposing patients to the onset of cardiac arrhythmia (125). Notably, the elevated release of proinflammatory, pro-fibrotic and prooxidant molecules in NAFLD patients such as cytokines, hepatokines, adipokines and gut-derived factors, were also able to change myocardial electrical properties and structural substrates, increasing the vulnerability of arrhythmias (126). For instance, proinflammatory cytokines, namely TNF-α, IL-1 and IL-6, can induce myocardial and atrial remodeling by modulating potassium and calcium homeostasis, prolonging action potential duration and QTc interval duration and reducing the expression of sarco-endoplasmic reticulum Ca2+ ATPase 2a (SERCA2a) and connexin-40 (125, 126). Additionally, proinflammatory cytokines also promote myolysis, cardiomyocyte apoptosis and myocardial fibrosis, and affect the expression and function of specific connexin-formed channels that possibly contribute to the development of re-entry arrhythmias. Conflicting results on the association between adipokines and AF were often reported, but a recent meta-analysis of 34 studies by Agbaedeng et al. found that adipokines, principally adiponectin and resistin, were associated with the risk of AF (127). Moreover, a Mendelian randomization study revealed that resistin levels was positively correlated with AF risk (OR = 1.09, 95% CI: 1.04–1.13) (128). Mechanistically, adipokines promoted systemic oxidative stress and inflammatory states that were involved in the occurrence and development of AF (127). Among gut-derived factors, TMAO not only enhanced susceptibility to AF by exacerbating cardiac autonomic nerves activity and promoting inflammatory responses, but also promoted ventricular arrhythmia via activating cardiac SNS (129). Furthermore, higher TMAO levels were associated with increased risk of adverse cardiovascular events in AF subjects (130). LPS had shown to up-regulate NLRP3 inflammasome expression and increase atrial fibrosis, contributing to the development of AF. Zhang et al. revealed that a selective inhibitor of the NLRP3 inflammasome (MCC950) can reduce atrial fibrosis and AF susceptibility by inhibiting inflammasome (131). Contrary to other factors, emerging lines of evidence showed that FGF21 had a beneficial effect on CVD that can protect against atrial remodeling by reducing oxidative stress and preventing atrial fibrosis, which provide new strategies for AF (132). Furthermore, SCFAs and bile acids also alleviated AF development via regulating GPR43/NLRP3 signaling pathway and promoting apoptosis of atrial myocytes, respectively (133). Strikingly, in subjects with NAFLD, the study on the role of hepatokines, adipokines and gut-derived factors in cardiac arrhythmia is scarce, and the above evidence is mainly based on general population or diabetes mellitus patients, thereby further research is needed to verify these findings.

### 3.4. Heart failure

Heart failure (HF), the end-stage of CVD, is a not uncommon but life-threatening manifestation of cardiac involvement. Growing evidence supported that NAFLD and HF often coexisted as they shared similar pathophysiological features and a variety of cardiometabolic risk factors (134). Moreover, there was a strong association between NAFLD and increased risk of new-onset HF, and this risk increased with the severity of NAFLD, especially in the fibrotic stage (134). A meta-analysis of 11 longitudinal cohort studies with a median follow-up of 10 years disclosed that NAFLD was associated with a 1.5-fold increased risk of new-onset HF, regardless of the presence of diabetes, hypertension, adiposity and other common cardiovascular risk factors (135). Simultaneously, in-hospital mortality of primary HF with preserved ejection fraction (HFpEF) and reduced ejection fraction (HFrEF) was higher in patients with NAFLD compared with those without NAFLD (136). In addition to the comorbidity of cardiovascular risk factors, several putative mechanisms that contribute to the relationship between NAFLD and increased risk of HF have been proposed. First, IR, disturbance of glucose and lipid metabolism were the crucial pathophysiological characteristics of NAFLD, which may eventually lead to the decrease of myocardial energy metabolism, cardiac dysfunction and remodeling (126, 134). Second, the activation of chronic systemic inflammation and mitochondrial dysfunction facilitated the production of higher levels of ROS and proinflammatory cytokines (e.g., IL-1β, IL-6 and TNF-α), which were the critical mediators of HF progression (126). Third, increased activity of RAAS and SNS, imbalance with angiotensin-converting enzyme 2 (ACE2)/Ang 1–7 axis, and altered expression of gut microbiota-derived metabolites were also related to HF development in NAFLD patients (82). It was worth mentioning that expression change of adipokines, hepatokines and gut-derived factors also were involved in the development of HF. For example, resistin, a new metabolic marker, was associated with the incident and severity of HF and can predict HF prognosis. Furthermore, for each standard deviation (7.45 ng/ml) increased in resistin, the risk of developing new-set HF elevated by 26% (137). These relationships may be induced by IR, abnormal glucose metabolism, inflammation, and depressed cardiomyocyte contractility. A prospective observational study demonstrated that higher Sep levels were also connected with hepatic hypoperfusion and predicted adverse outcomes in HF (138). A recent Mendelian randomization study indicated that RBP4 levels were causally associated with an increased risk of HF (OR = 1.14, 95% CI: 1.02–1.27), but the underlying pathogenesis still needs to be further explored (128). Elevated TMAO concentrations enhanced the risk of HF by increasing myocardial contractility and calcium flux in a dose-dependent manner, exacerbating myocardial fibrosis and accompanying diastolic dysfunction (139). Moreover, high TMAO levels were related to disease severity and poor prognosis in patients with HF (140). Notably, although there are few studies focusing on the association between SCFAs and HF, limited evidence suggests that SCFAs have protective effects on intestinal mucosal barrier. The depletion of SCFAs would lead to intestinal barrier disruption, which facilitates LPS and proinflammatory cytokines transport into systemic circulation and aggravates cardiac remodeling by modulating CD4+ T cells, ultimately resulting in HF (141). Altogether, the above-mentioned results indicate that metabolic organ-secreted factors expression levels are correlated well with the risk and prognosis of HF, but the exact mechanism requires further elucidation.

It is worth mentioning that altered expression levels of metabolic organ-secreted factors are associated with the pathogenesis of NAFLD and CVD, but it is relatively difficult to determine the total effect of these metabolic organ-secreted factors in the occurrence and development of cardiovascular events, given the interactions of multiple organs throughout the body. Secretome analysis and system biology approach may provide better insights into the role of metabolic organ-secreted factors, which still need further study.

## 4. Discussion

CVD remains the leading cause of death worldwide over the past 10 years. NAFLD patients have a significant risk of CVD compared with the general population, and the proportion of CVD-related deaths in NAFLD individuals is higher than that of liver-related deaths. Thus, a comprehensive and detailed assessment of CVD risk in NAFLD patients is essential for improving clinical outcomes. To date, the exact pathogenesis of the increased risk of CVD in NAFLD patients remains unclear. Increasingly more data demonstrated that metabolic organ-secreted factors (e.g., hepatokines, adipokines, cytokines, chemokines, EVs, gut-derived factors, etc.) were closely connected with the development of CVD that may be contributed to by systemic inflammation, lipid disturbances, IR, RAAS and vessel dysregulation, oxidative stress, and altered host's metabolic phenotype. However, whether these metabolic organ-secreted factors can be used as diagnostic and prognostic markers or as therapeutic targets of CVD in NAFLD subjects is not yet fully understood and require further investigation.

It is worth noting that, in addition to the aforementioned metabolic organ-secreted factors, increasing studies have begun to focus on the relationship between myokines, uric acid (UA) and metabolic diseases. Myokines are a class of cytokines or peptides synthesized secreted by myocytes in muscle tissue, which are implicated in autocrine regulation of muscle metabolism as well as in the paracrine/endocrine regulation of other tissues, such as adipose tissue and liver. Irisin, IL-6 and myostatin are the most extensively studied myokines in NAFLD, and serum irisin and IL-6 levels and muscle irisin and IL-6 expression are significantly elevated in exercised NAFLD or NASH mouse models (59, 142). Limited evidence proposed that irisin had the properties of promoting muscle hypertrophy, enhancing insulin sensitivity, alleviating hepatic steatosis and anti-inflammation, and improving metabolic syndrome and CVD, but the underlying mechanism still needs further characterization (142, 143). IL-6 derived from myocytes has been reported to mediate glucose homeostasis, white adipose tissue lipolysis, and inflammatory reaction, as it stimulates the release of IL-10, which creates an anti-inflammatory environment (142). Myostatin is an inhibitor of muscle growth and a promoter of liver fibrosis, but its levels are reduced in NAFLD patients with the practice of exercises (144). Moreover, decreased myostatin could delay the progression of NASH by reducing fat accumulation and inflammation, and promoting FFA redistribution (144). Therefore, physical activity plays a relevant role as a therapeutic target. In all, myokines are also involved in the pathogenesis of NAFLD. Besides, UA is an end product of purine metabolism, and hyperuricemia is mainly caused by purine metabolism or UA excretion disorders. A growing body of research revealed a correlation between serum UA levels and NAFLD, and UA was an independent risk factor for NAFLD (145, 146). Moreover, the risk of NAFLD in subjects with hyperuricemia was significantly higher than individuals with normal UA level (OR = 1.97, 95% CI: 1.69–2.29) (147). The potential mechanism may be contributed to IR, inflammation, oxidative stress, increased reactive oxygen species (ROS), and fat accumulation in hepatocytes (148). However, the study on the association between myokines and hyperuricemia linking NAFLD and CVD is rare, which still needs further exploration.

Due to the complexity of the pathogenesis of NAFLD, there are currently no FDA-approved drugs for the treatment of this disease. Comprehensive lifestyle modifications and increased public awareness are likely the most cost-effective ways to contain NAFLD pandemic. Notably, the debate on the causal relationship between CVD and NAFLD continues, and whether metabolic organ-secreted factors are independent risk factors for CVD in NAFLD patients is still unknown. Further conclusive evidence is needed to support either association, which plays a crucial role in the decision whether to recommend early screening, close follow-up, and aggressive intervention for high-risk population in clinical practice.

## Author contributions

LQ, CW and JC contributed to the conception and design of the study. LQ, JW, XS, XH, WH and CW performed literature search and data collection. LQ and JC wrote the draft of the manuscript and performed the initial revision. All authors contributed to the article and approved the submitted version.
